# An Automatic Detection Model of Defects in Pipelines in Complex Environments

**DOI:** 10.3390/s26113418

**Published:** 2026-05-28

**Authors:** Shiyuan Zheng, Zhaochao Li

**Affiliations:** 1 School of Civil and Environmental Engineering, Hunan University of Technology, Zhuzhou 412007, China; zsssssyuan@stu.hut.edu.cn; 2 Intelligent Control of Safety and Risk for Existing Engineering Structures, Key Laboratory of Hunan Province, Hunan University of Technology, Zhuzhou 412007, China

**Keywords:** deep learning, YOLO, automatic detection, CNN, pipeline defects

## Abstract

Metal pipelines may have various defects due to long-term service, corrosion, external strikes, etc. Traditional closed-circuit television (CCTV) inspection techniques are capable of detecting these defects. However, substantial human resources are required and the detection results are subjected to human subjectivity. Thus, this study develops a deep learning-based intelligent defect detection model for metal pipeline images. CNNs (convolutional neural networks) are utilized to automatically extract defects, which may mitigate the interference of subjective factors and enhance the recognition capability of the defects in pipelines. The proposed model builds upon the original YOLOv8n model by incorporating the SCSA (Spatial and Channel Synergistic Attention) mechanism, LskBlock, and SlideLoss function, respectively. These enhancements improve the ability to detect small targets, increase recognition accuracy, and facilitate global optimization, respectively. The developed YOLO-LSS (YOLOv8n-LskBlock-SlideLoss-SCSA) model is compared with other deep learning models characterized by the following metrics: mAP50, mAP50:95, precision, recall rate, and F1-score, respectively. It is found that mAP50 achieves 79.05% (+2.86%), mAP50:95 53.7% (+7.19%), precision 81.6% (+5.30%), recall rate 75.5% (+2.90%), and F1-score 78.4 (+4.01), indicating that the proposed model effectively enhances the capability of detecting internal defects in pipelines.

## 1. Introduction

Metal pipelines are essential transportation tools to convey petroleum, natural gas, chemicals, and water in modern industries [1,2]. Pipeline transportation offers higher efficiency, lower operational costs, and less environmental pollution than road and rail systems [3]. However, the long-term deployment of pipelines may lead to critical defects, which commonly arise from changes in the surrounding environment or corrosion caused by the substances transported in the pipelines [4,5]. These defects jeopardize normal functionality and pose serious risks to public safety [6]. Thus, it is paramount to timely detect these defects [7]. Numerous techniques have emerged to detect surface defects of pipelines with the continuous advancement of sensor and computing technologies, including closed-circuit television (CCTV) [8], Non-Destructive Testing (NDT) [9], and Ground-Penetrating Radar (GPR) [10], etc. Commonly, NDT encompasses visual inspection, magnetic flux leakage testing [11,12], sonar testing [13], laser projection imaging, and pulsed eddy current testing [14].

Traditional detection techniques often compromise detection effectiveness due to the inherent properties of metal pipelines. CCTV remains the most widely applied detection technology because of its low cost. It involves operators maneuvering robots into pipelines to capture inspection videos, which are then interpreted and recorded by specialized operators to offer accurate assessments. This technique eliminates the laborers entering the pipelines during inspections. However, it still requires skilled professionals to operate equipment and interpret video footage. The involvement of humans introduces a substantial degree of subjectivity in defect assessment and may result in relatively low efficiency.

Recently, automated analysis has become a prevailing trend due to the limitations of manual interpretation of defect videos. Traditional computer vision techniques primarily include threshold segmentation [15] and edge detection [16]. Both approaches aim to extract significant features from images for subsequent processing, employ preprocessing methods to highlight critical information, and utilize variations in pixel grayscale values to identify image characteristics [17]. However, these techniques face considerable challenges in extracting defects in real-world scenarios, especially in some complex backgrounds of pipelines.

Convolutional neural network (CNN)-based technologies have been developed rapidly to detect defects [18] with the rapid advancement of artificial intelligence (AI). Currently, CNN-based algorithms are categorized into one-stage and two-stage detection algorithms. One-stage algorithms include SSD [19] and YOLO [20] series models, while two-stage algorithms encompass RCNN [21], Fast R-CNN [22], Faster R-CNN [23], and Mask R-CNN [24]. One-stage algorithms offer higher speed but lower accuracy in detecting defects. Conversely, two-stage algorithms achieve higher precision but lower speed. The internal defect requires a high level of real-time detection in pipelines, which is also a huge challenge to the present detection methods [25]. Therefore, this study improves the YOLO model, one of the single-stage detection algorithms, to balance the precision and speed in the detection of the pipelines.

Numerous studies have modified one-stage and/or two-stage detection algorithms to identify specific types of defects. Zhao et al. [26] accurately extracted defect features from images by introducing an iRMB module to their detection network based on YOLOv8. Xu et al. [27] incorporated a CA attention mechanism into the original YOLOv5 model and improved the loss function, achieving effective recognition of pipeline weld seams. Klusek et al. [28] utilized YOLOv2 as their detection network deployed on embedded devices. This network covered both the classification and detection of pipeline defects. Wang et al. [29] employed Faster R-CNN for defect detection and proposed an algorithm for tracking defect identification in continuous CCTV video tasks, achieving an Identity F1-Score (IDF1) of 57.4%. However, there remains room for improvement in precision. Yin et al. [30] developed a video interpretation algorithm for sewer pipelines (VIASP) based on the YOLOv3 model that extracts key information from videos and enables automatic defect labeling, ultimately outputting evaluation reports in tabular text format. However, the processing speed is slow and the results are unsatisfactory. Chen et al. [31] integrated EfficientVit as a feature extraction network for defects in the original YOLOv8 model, which significantly reduces the number of parameters and effectively improves the detection accuracy of defects. Lv et al. proposed an adaptive multi-scale detection transformer (AM-DETR) to effectively identify surface defects against complex backgrounds. In the specific field of pipeline inspections, researchers are actively exploring hybrid models and attention mechanisms. Furthermore, another study by Chen et al. designed a cascaded deep learning approach combining YOLOv5 and Vision Transformer (ViT) to accurately detect and classify pipeline defects from magnetic flux leakage (MFL) data. Additionally, recognizing the computational and environmental constraints of practical pipeline inspections, lightweight models have garnered considerable attention. Li et al. developed a lightweight YOLOv8-based model incorporating Squeeze-and-Excitation Version 2 (SEV2) and GhostConv modules, which successfully enhanced feature extraction and crack detection accuracy in low-light and complex pipeline backgrounds. While these studies have made substantial progress, accurately detecting small-sized defects in noisy, low-contrast CCTV images while maintaining real-time inference speed remains a challenging task.

This study modifies the YOLOv8 framework to effectively recognize defects in pipelines. Experimental results indicate that the improved model substantially enhances the performance in automatically detecting internal defects (e.g., corrosion, deposition, oxide shedding, and penetration) in metal pipelines. The main contributions of this study are below:

(1) A novel YOLOv8n-LskBlock-SlideLoss-SCSA model is proposed and public datasets, which contain defect samples under extreme low-light conditions and complex environments, are utilized to accurately detect defects in metal pipes.
(2) Compared to existing defect detection algorithms in the pipeline, the proposed method is capable of simultaneously achieving high accuracy and real-time detection of various defects in the pipeline. Additionally, the model is lightweight, facilitating deployment on terminal devices and adequately addressing practical operational requirements to a certain extent.
(3) Extensive experiments were conducted and the superiority of the proposed model was verified by comparing it with the current mainstream models.

The remainder of this paper is organized as follows: Section 2 offers a detailed introduction to the architecture of the proposed YOLO-LSS model. Section 3 outlines the comprehensive experimental setup, encompassing dataset preparation, image augmentation techniques, experimental equipment, and model evaluation metrics. Section 4 presents a detailed analysis of the comparative and ablation experimental results. Finally, Section 5 summarizes the entire work and discusses potential directions for future study.

## 2. A Defect Detection Method Based on YOLO

### 2.1. YOLOv8 Model

The YOLO model was initially proposed by Redmon et al. [32], with its most innovative aspect transforming the object detection problem into a regression problem. This allows for the simultaneous prediction of multiple bounding boxes and class labels within an image using a single neural network. Different from the traditional object detection algorithms using stepwise region proposal [33], the YOLO model significantly enhances detection speed. However, the first-generation version also exhibited drawbacks such as poor localization accuracy, insufficient capability in detecting small objects, and weaknesses in handling complex scenes. To address these limitations, subsequent versions of the YOLO series have undergone continuous improvements and optimizations [34,35,36,37,38]. This paper focuses on optimizing and improving the YOLOv8n model to enhance algorithmic precision. Section 4.1 elaborates on the rationale for selecting the YOLOv8 model for modification.

**Figure 1:**
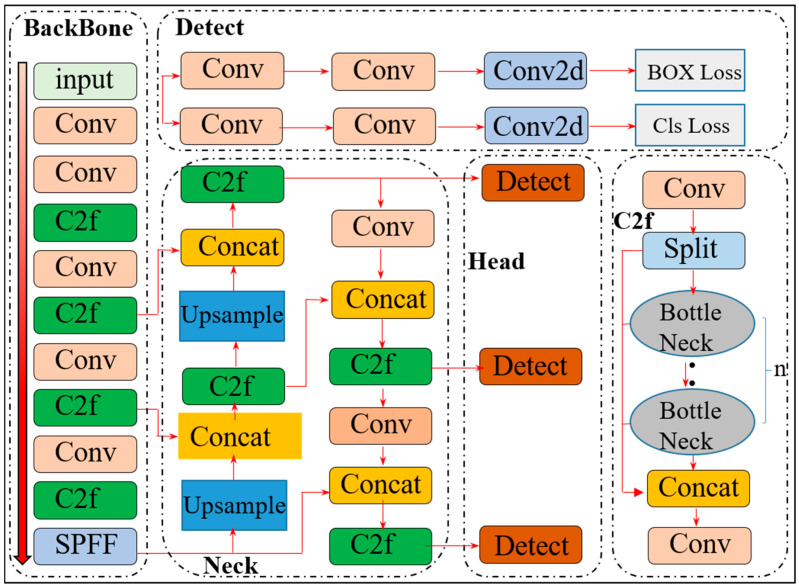
Network of YOLOv8 model.

YOLOv8 is a next-generation open-source object detection framework launched by the Ultralytics team in 2023, with its architectural diagram illustrated in Figure 1. As one of the most practical technical iterations within the YOLO series, it encompasses core functionalities, such as object detection, instance segmentation, and real-time tracking scenarios, etc. The structure of the YOLOv8 model consists of three essential components: a backbone network, neck network, and head network. At the input stage, data preprocessing is performed by resizing all input images to 640 × 640 pixels followed by applying Mosaic augmentation to facilitate subsequent feature extraction processes. Effective integration occurs across multi-scale feature maps to construct a feature pyramid when different levels of feature maps are collected by the feature fusion module.

The neck network combines concepts from the Path Aggregation Network (PAN) and Feature Pyramid Network (FPN), aiming to enhance robustness and learning performance by improved representation of various features. The head component is responsible for predicting information regarding target classes, locations, and confidence scores, among other details. Compared to earlier versions, YOLOv8 improves the accuracy of the bounding box and addresses issues associated with smaller models in detection tasks through innovations such as the distribution focal loss (DFL) function and Complete Intersection over Union (CIoU).

### 2.2. Present Improvement

#### 2.2.1. Introduction of the SCSA-Improved Feature Extraction Module

The attention mechanism (AM) is commonly used to optimize the performance of CNNs, significantly enhancing the model’s feature understanding capability. The AM focuses selectively on key features to improve the detection capability of the model by assigning different weights to input information and performing weighted summation. Based on the scope of its application, the AM is classified into three types: spatial AM, channel AM, and hybrid AM.

**Figure 2:**
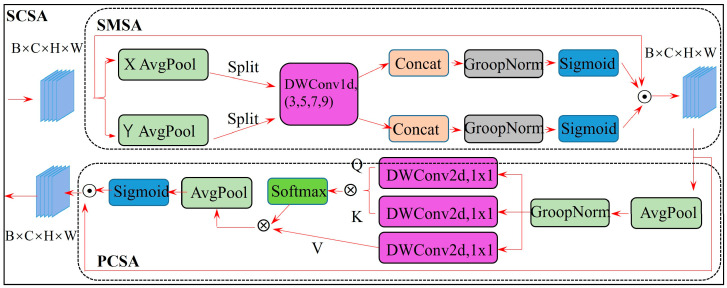
Structure of spatial and channel collaborative AM.

The present work proposes an improved SCSA (Spatial and Channel Synergistic Attention) [39] mechanism, which is an innovative hybrid AM that combines the spatial and channel AMs. It achieves cross-dimensional feature enhancement by decoupling multi-semantic information from both spatial and channel dimensions. The core of SCSA consists of two modules: Shared Multi-semantic Spatial Attention (SMSA) and the Progressive Channel Self-Attention Mechanism (PCSA). The structure of the SCSA module is shown in Figure 2.

The SMSA module processes the input feature map in three distinct steps. First, the feature map undergoes vertical and horizontal average pooling along the height and width dimensions, respectively, resulting in two single-column sequence features. Each of these sequence features is then partitioned into four groups of independent sub-features. Subsequently, SMSA employs multi-scale depth-shared 1D convolutions to extract spatial features across different semantic levels. Finally, group normalization (GroupNorm) [40] is applied to preserve the independence of the sub-features, thereby generating discriminative spatial prior information.

The PCSA module is responsible for extracting channel attention, and its process is also divided into three steps. Initially, the feature map output from the SMSA module undergoes average pooling and group normalization, leading to a preliminarily compressed feature map. Following this, three sets of 2D (two-dimensional) depthwise convolutions are applied to extract feature vectors and compute attention weights. Ultimately, an average pooling operation and activation function is employed to generate channel attention information.

#### 2.2.2. Large Selective Kernel Network (LskBlock)

Pipeline defect detection faces numerous significant challenges, including poor image quality, diverse defect types, complex environmental interference, stringent real-time requirements, and the need for algorithmic robustness. To address these challenges, this study incorporates the large selective kernel network (LSKNet) [41]—a novel attention mechanism—into the detection head of the YOLOv8 architecture. LSKNet explicitly constructs multi-scale features with large receptive fields by decomposing large convolutional kernels into a sequence of progressively expanded depthwise separable convolutions. Moreover, it introduces, for the first time, a spatially selective mechanism that adaptively integrates contextual information from varying spatial ranges based on the input feature representation.

**Figure 3:**
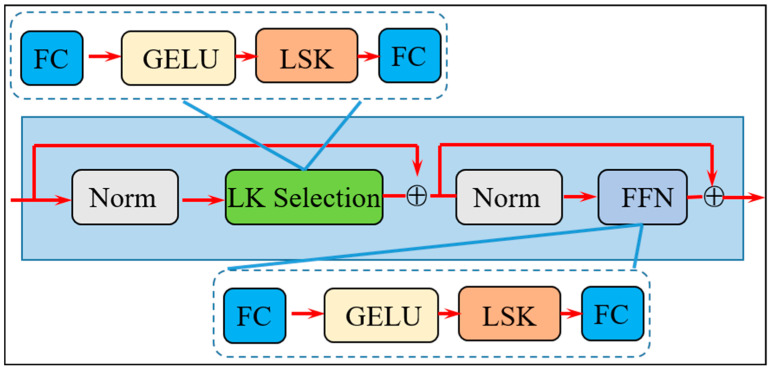
LskBlock structure diagram.

As illustrated in Figure 3, the LSK block comprises two primary sub-blocks: the Large-Kernel Selection (LK Selection) module and the Feed-Forward Network (FFN) sub-block. The LK Selection module employs large-kernel convolutions to capture extended spatial dependencies—a capability particularly beneficial for detecting pipeline defects that may span multiple scales. Specifically, this module utilizes a sequence of depthwise convolutions with varying kernel sizes and dilation rates, effectively enlarging the receptive field without incurring substantial computational overhead.

Pipeline defects often appear across diverse scales and against complex backgrounds. In the proposed YOLO-LSS model, the LSK block is integrated into the detection head at three distinct feature pyramid levels—P3, P4, and P5—corresponding, respectively, to small-, medium-, and large-scale defect detection. This multi-scale integration enables each detection branch to independently optimize its receptive field according to the characteristic size of its target objects, thereby allowing the model to simultaneously capture fine-grained local defect features and broader contextual information.

#### 2.2.3. SlideLoss Function Enhancement

The SlideLoss function [42], is an advanced loss function designed for deep learning-based object detection tasks. It addresses the critical challenge of class imbalance between easy and hard training samples by introducing an IoU-based adaptive weighting mechanism. In the YOLO-LSS model, SlideLoss serves as a loss modulator applied to the classification loss of YOLOv8. This enables dynamic reweighting of the classification loss according to the quality of prediction, thereby enhancing the model’s ability to learn effectively from samples of varying difficulty.

**Figure 4:**
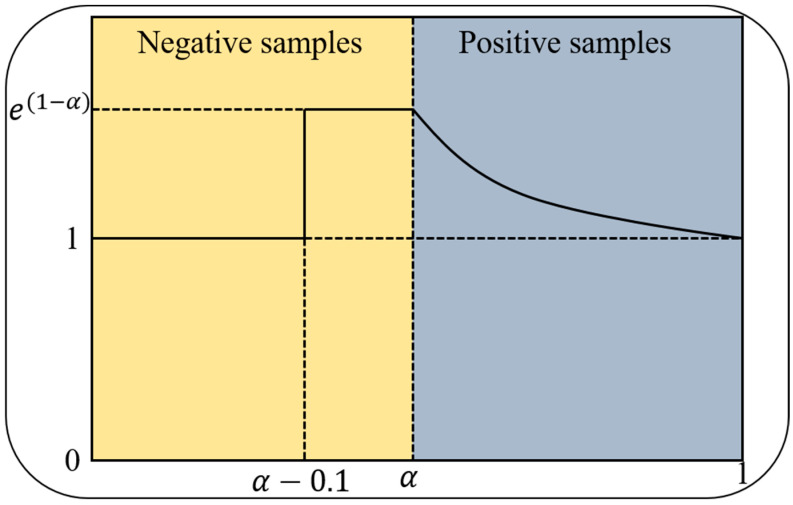
Slide weighting function.

The core of SlideLoss lies in its adaptive weighting function, which applies piecewise weighting based on the degree of alignment between predictions and ground-truth labels, as shown in Figure 4:

(1) $$f\left(x\right)=\left\{\begin{array}{c} 1,x\leq \alpha -0.1\\ e^{1-\alpha },\alpha -0.1<x<\alpha \\ e^{1-x},x\geq \alpha \end{array}\right.$$

where x denotes the predicted confidence score or the IoU value; α is a threshold parameter, set to 0.5 by default in implementation. When α < 0.2, it is automatically clamped to 0.2 to ensure training stability.

Applying SlideLoss to the classification loss increases the loss weight for negative samples whose confidence scores lie near the classification decision boundary—thereby encouraging more precise discrimination between background and foreground objects. Conversely, it reduces the loss weight for high-confidence, easy positive samples to prevent them from dominating the optimization process. Finally, it assigns moderate yet elevated weights to samples with confidence scores in the boundary region—samples that typically correspond to ambiguous or challenging cases, such as subtle defects or occluded objects.

In pipeline defect detection tasks, the internal pipeline environment is highly complex, rendering the discrimination between defects and the background inherently challenging. SlideLoss addresses this challenge by adaptively reweighting classification losses, thereby enabling the model to learn discriminative defect features more effectively. Pipeline images captured under low-light conditions typically contain substantial noise; to mitigate its adverse impact on classification performance, SlideLoss is specifically designed to down-weight evidently background-dominated regions. Moreover, defect boundaries are often ambiguous and ill-defined; SlideLoss enhances learning emphasis on boundary-proximal samples, thereby improving the precision of boundary-aware classification.

Furthermore, SlideLoss synergizes with other architectural innovations in the YOLO-LSS framework: after the SCSA attention mechanism identifies salient defect regions, SlideLoss refines the classification learning process within those regions. The LskBlock, leveraging an enlarged receptive field, captures multi-scale contextual information, providing SlideLoss with more accurate and semantically rich feature representations for reliable sample hardness estimation. Collectively, these components establish a holistic, end-to-end optimization pipeline—from hierarchical feature extraction and attention-guided region focusing to adaptive loss calibration—thereby achieving comprehensive performance enhancement.

#### 2.2.4. The YOLO-LSS Model

**Figure 5:**
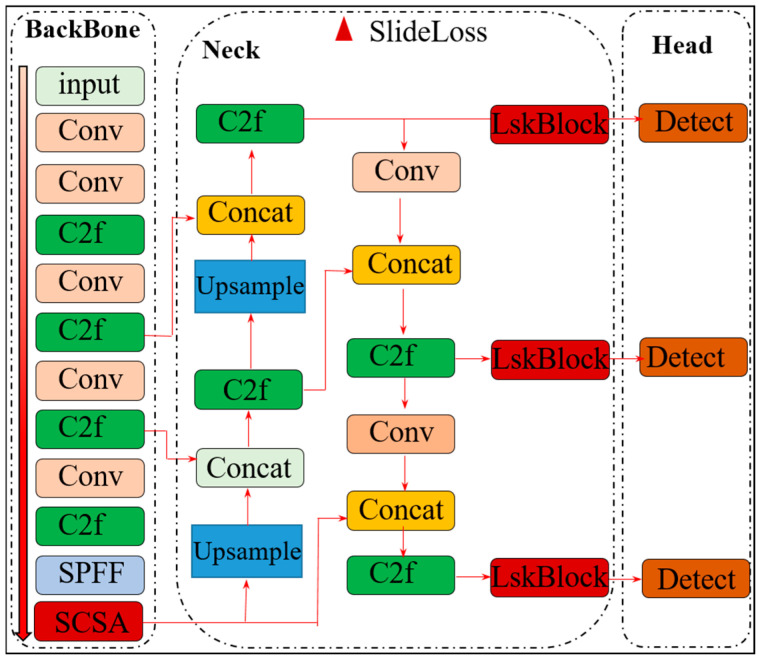
The architecture of the proposed YOLO-LSS model.

The optimized architecture of the YOLO-LSS model is illustrated in Figure 5. To address the detection challenges in complex pipeline environments, this study restructures the YOLOv8 framework across three dimensions. In the feature extraction stage, the SCSA module is introduced. By synergistically optimizing feature representations in both the channel and spatial dimensions, this module effectively suppresses background noise interference, significantly enhancing the model’s capability to extract purified key defect features. In the neck network, the LskBlock is integrated into the feature fusion mechanism. Leveraging its multi-branch large-kernel structure, the model can dynamically adjust its spatial receptive field, thereby effectively adapting to the drastic scale variations in pipeline defects and ensuring the accurate detection of both minute corrosion spots and extensive cracks. Finally, in the objective optimization stage, the SlideLoss mechanism is adopted to improve the classification loss function. By assigning higher gradient weights to hard examples via a dynamic sliding threshold, it effectively alleviates the gradient imbalance between massive easy background samples and blurred hard samples.

During the forward propagation phase, SCSA and LskBlock function interactively: SCSA first filters background interference by reweighting network features, and LskBlock subsequently receives these initially denoised features to adaptively adjust the receptive field. This consecutive process effectively extracts the topological features of multi-scale defects. During the backpropagation phase, SlideLoss tightly couples the objective optimization with these frontend modules. Because the pipeline dataset contains numerous easy background samples, SlideLoss specifically assigns larger error weights to hard samples near the decision boundary. These amplified loss gradients are directly backpropagated to SCSA and LskBlock, explicitly guiding their parameter updates and forcing their attention weights and spatial selection mechanisms to lean toward hard-to-distinguish complex defects.

From the perspective of architectural completeness, these three components establish a highly effective synergistic combination to address the critical challenges of underground pipeline detection. The necessity of each module is logically clear: without SCSA, LskBlock would process features containing substantial noise, making its dynamic spatial selection mechanism highly susceptible to failure; without LskBlock, the network would rely on a static receptive field, limiting its adaptation to geometric variations in multi-scale defects; and without SlideLoss, gradient updates would be dominated by easy samples, failing to effectively drive the frontend modules to optimize for challenging blurred samples. Therefore, the integration of SCSA, LskBlock, and SlideLoss constitutes a logically rigorous synergistic mechanism: feature denoising, scale adaptation, and hard-example gradient modulation. The quantitative results of the ablation study further confirm that the absence of any single component leads to performance degradation, demonstrating the significant contribution of each module and the overall effectiveness of the proposed architecture.

## 3. Experimental Setup and Evaluation Metrics

### 3.1. Dataset and Image Augmentation

The training images in this experiment are sourced entirely from a pipeline dataset provided by the open-source platform Roboflow Universe—specifically, the pipe-v7 dataset. The images in the test set are classified and annotated by several experienced pipeline defect repair experts, forming a proprietary dataset. In this dataset, metal pipelines are mainly utilized for transporting liquids such as water, crude oil, refined oil, and liquefied natural gas. Most of the images typically exhibit low contrast and high noise levels since these images are captured under low-light conditions, which results in blurred details that adversely affect the recognition accuracy of subsequent experimental models. All collected images are automatically adjusted to 640 × 640 pixels before experiments to ensure input consistency and support the learning of scale-invariant features within the scope of our dataset. These images are also subjected to grayscale processing.

Furthermore, several image enhancement techniques are applied: (1) random horizontal flipping of half of the images; (2) random vertical flipping of half of the images; and (3) random cropping applied to 20% of the images within the dataset. The original set is expanded from 2584 to 7752 images by using the above image augmentation methods. Gaussian noise is introduced at a rate of 5% to some images to simulate artifacts caused by insufficient light sensitivity under low-light conditions. This addition increases image complexity and compels the present model to learn effective features amidst more disruptive environments, which improves the recognition accuracy of the model under simulated noisy conditions within the test set.

This study utilizes a dataset that is functionally divided into three independent subsets: the training set, validation set, and test set. The segmentation is outlined in Table 1. The training set (7752 images) is employed for optimizing model weights, while the validation set (259 images) monitors potential overfitting and evaluates generalization capabilities. The test set (260 images) serves as the final assessment. All samples are processed by using the YOLO annotation format.

**Figure 6:**
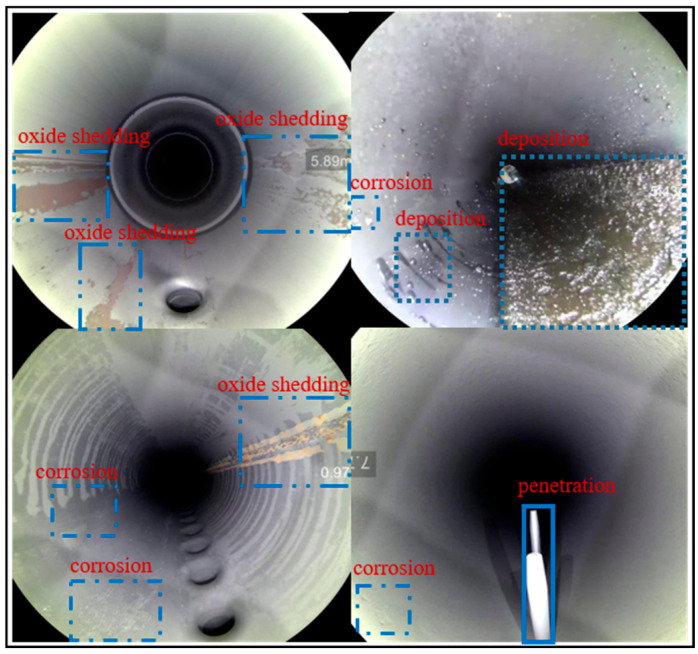
Four types of defects in the inner wall of metal pipelines.

The four types of defects are illustrated in Figure 6. The first type is corrosion (dotted lines). This occurs when chemical reactions between the pipeline and the medium or external environment lead to localized damage on the metal surface. The second type is deposition (dashed lines). This may be induced by the accumulation of solid materials on the inner wall of the pipeline or blockage over time. The third type is oxide shedding (double-dotted lines). Detachment of oxide layers may cause surface roughness and promote further corrosion in high-temperature environments. The last type is penetration (solid lines). This is caused by internal blockages or cracks within the pipeline, leading to fluid leakage or loss. This dataset has been meticulously designed with a strict sample ratio across categories. In such a circumference, a comprehensive representation of each category is guaranteed to achieve a scientific partitioning of training samples, validation samples, and test samples. This approach effectively supports the robust training process of deep learning models.

### 3.2. The Experimental Equipment

The images in the dataset were collected by a Hikvision DS-2CD2087G2-L camera with a 1/1.2-inch progressive-scan CMOS sensor. This camera is capable of maintaining clear image quality even in low-light or dark environments. The specific hardware and software configurations for training and testing are listed in Table 2; the operation system is Windows 10 Professional Edition. The deep learning framework is PyTorch version 2.3.1, along with CUDA version 12.1, and Python version 3.8. The CPU is Intel Core i5-13490F, and the GPU is RTX 3070 Ti with a memory capacity of 8 GB. The training parameters are outlined in Table 3. Adam was selected as the optimizer, with an initial learning rate of 0.01, momentum at 0.937, and decay factor at 0.0005. The input image dimensions are specified as 640 × 640 pixels, with a batch size of 16 and a total training epoch of 300.

### 3.3. Model Evaluation Indicators

This experiment uses four metrics, mean Average Precision (mAP), precision (P), recall (R), and F1-score, to assess the model. mAP is the average detection accuracy of all categories, calculated from precision, recall, and AP. The definitions of the above metrics are as follows:

(2) $$AP=\int_{0}^{1}PdR$$

(3) $$mAP=\frac{\sum_{i=1}^{N}AP_{i}}{N}$$

where mAP is the most widely adopted metric to assess the model by integrating precision and recall values in multiple confidence thresholds. This metric systematically evaluates detection accuracy across multiple object categories. mAP is derived from the average of individual class-specific AP values, as shown in Equation (3). A higher value of mAP indicates superior detection performance across all target categories. The other metrics are calculated as follows:

(4) $$Precision=\frac{TP}{TP+FP}$$

(5) $$Recall=\frac{TP}{TP+FN}$$

(6) $$F1-score=\frac{2\times Precision\times Recall}{Precision+Recall}$$

Precision, formally defined as the ratio of true positive instances to all positively predicted samples (specifically referring to detected objects in computer vision tasks), serves as a critical quantitative metric for evaluating classification in machine learning (ML) systems. In the framework of object detection, precision quantification provides essential insights to minimize false alarms and maintain effective capabilities of target recognition. The recall rate refers to the proportion of correctly predicted positive samples identified by the model out of all actual positive samples. The F1-score is the harmonic mean of precision and recall, serving as a comprehensive metric that considers both aspects. A higher F1-score indicates a better balance between precision and recall, indicating that the model effectively reduces false positives when it detects more targets. In this context, AP denotes the area enclosed by the P-R curve and the coordinate axes; N represents the number of target categories to be detected; and TP, FP, and FN refer to true positives, false positives, and false negatives, respectively.

In addition to the aforementioned accuracy metrics, Frames Per Second (FPS) is employed to evaluate the real-time processing capability and inference efficiency of the model. The calculation formula is defined as follows:

(7) $$FPS=\frac{N_{image}}{T}$$

where $N_{image}$ denotes the total number of image frames processed by the model, and T represents the total time (in seconds) required to process these frames. A higher FPS value indicates a faster detection speed, which is a critical hardware deployment requirement for real-time pipeline inspection tasks.

## 4. Results and Discussions

### 4.1. Refinement of Benchmark Algorithm Prior Experiments

Several preliminary experiments are conducted on various YOLOv8 algorithms to determine the optimal version for detecting the defects of pipelines. The experimental results are depicted in Table 4. One may see the YOLOv8n model has the lowest computational requirements and achieves the fastest detection speed. However, its accuracy and other metrics are lower than the other models. In contrast to YOLOv8n, models such as YOLOv8s, YOLOv8m, YOLOv8l, and YOLOv8x demonstrate superior performance, particularly in detection accuracy. These models significantly enhance the detection capabilities of defects by increasing both the depth and width of the neural networks. Nonetheless, these models are more complex and demand more computational power, resulting in a marked lower inference speed than the YOLOv8n model. Pipeline inspection requires high real-time performance, and detection speed is a key factor in practical scenarios. Thus, the YOLOv8n model is selected as the benchmark in the present study.

### 4.2. Refinement and Validation

**Figure 7:**
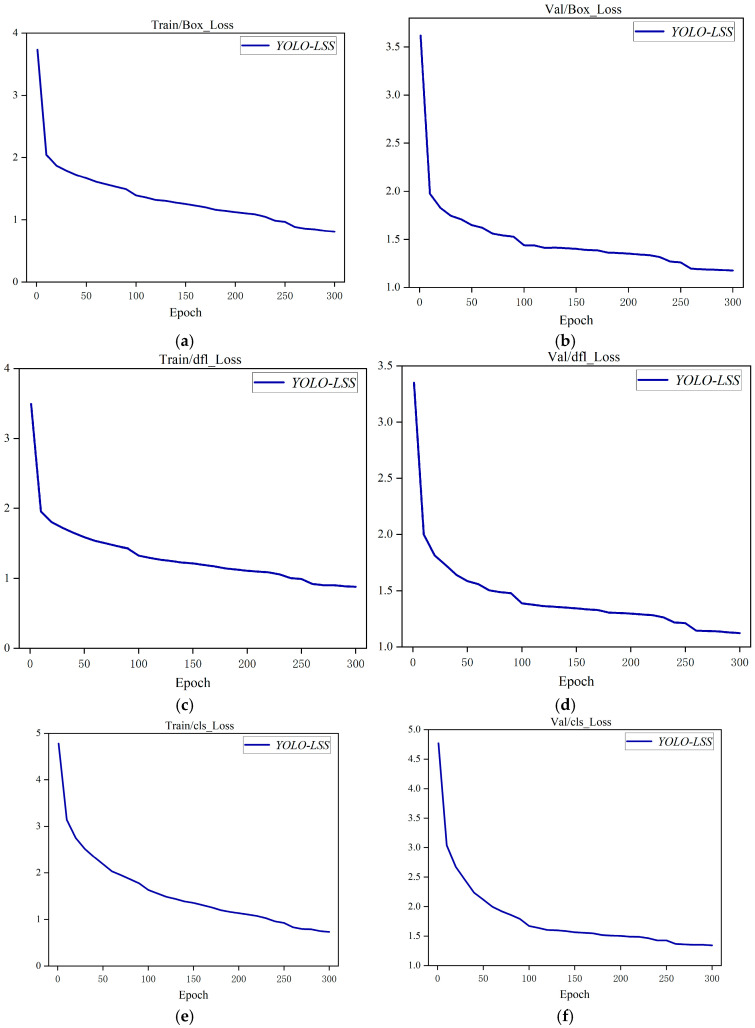
The training curves for: (**a**) box loss in the training dataset; (**b**) box loss in the valid dataset; (**c**) dfl loss in the training dataset; (**d**) dfl loss in the valid dataset; (**e**) cls loss in the training dataset; and (**f**) cls loss in the valid dataset.

The improved YOLOv8n-LskBlock-SlideLoss-SCSA model achieves a precision of 81.5% on the validation set, underscoring its high accuracy in detecting the defects of pipelines. Figure 7 provides a clear illustration of the loss values corresponding to box loss, classification loss, and distribution focal loss, respectively. Box loss is primarily utilized for bounding box regression tasks in object detection and measures the discrepancy between predicted boxes and ground truth boxes. In contrast, classification loss quantifies the likelihood that identified objects belong to specific categories. Distribution focal loss specifically addresses class imbalance issues, particularly in object detection scenarios where there is an imbalance between positive and negative samples. Focal loss enables the model to focus more on challenging-to-classify samples, thereby enhancing the overall performance of the model. It is observed that all three types of losses gradually decrease and ultimately stabilize at lower levels, highlighting the validity of the improved model.

**Figure 8:**
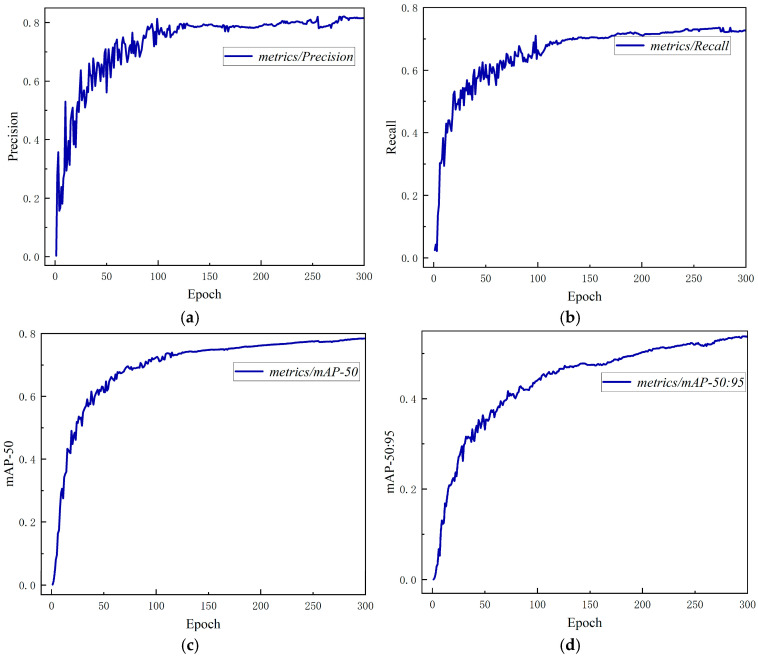
Training results for: (**a**) precision, (**b**) recall, (**c**) map50, and (**d**) mAP50–95.

Figure 8 illustrates the variations in four metrics (precision, recall rate, mAP50, and mAP50-95) for 300 training iterations in the enhanced model. Notably, all four metrics enhance remarkably within the first 50 epochs, and then increase slowly until convergence occurs at epoch 143. This trend indicates that the performance of the proposed model improves as the epoch increases, and the proposed model is appropriate to detect the defects of pipelines.

**Figure 9:**
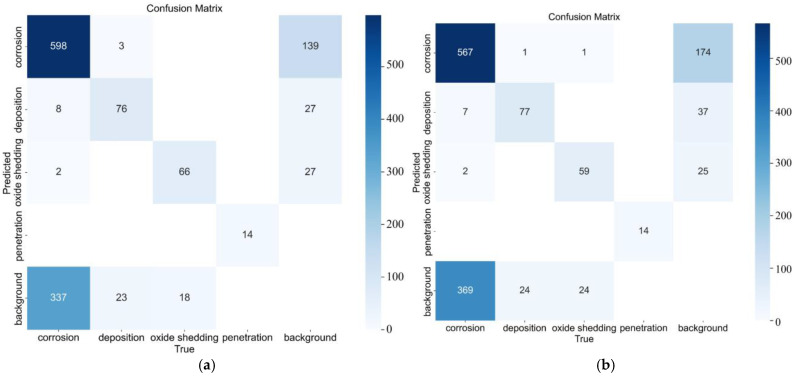
Confusion matrices of (**a**) YOLO-LSS model, (**b**) YOLOv8n model.

The confusion matrix of the improved model is shown in Figure 9a, and that of the original model is shown in Figure 9b. The confusion matrix provides insights into the number of true positives, true negatives, false positives, and false negatives for each category, thereby offering a deep view of the model’s classification accuracy.

It can be seen from Figure 9 that, compared with the original YOLOv8n model, the YOLO-LSS model effectively reduces the false positive rate. For instance, the number of background instances incorrectly predicted as corrosion and deposition decreased from 174 and 37 to 139 and 27, respectively. Concurrently, the true positive detections increased (e.g., correctly identified corrosion instances rose from 567 to 598), and the false negatives decreased. This simultaneous reduction in misclassifications robustly verifies that the improved YOLO-LSS model possesses superior feature discrimination and more precise localization capabilities when identifying complex pipeline defects.

To further understand the model’s limitations in complex environments, a theoretical analysis of the misdetection cases (false positives and false negatives) was conducted. False positives primarily occur when background artifacts—such as water reflections, uneven illumination, or inherent non-structural rust stains on the pipe wall—exhibit high inter-class visual similarity to actual defects. These artifacts generate strong local gradient responses in the convolutional feature maps that closely mimic the textural and geometric patterns of ‘corrosion’ or ‘deposition’, leading to misclassification. Conversely, false negatives are predominantly associated with ‘oxide shedding’ and extremely small-scale defects. From a computer vision perspective, ‘oxide shedding’ often presents exceptionally low contrast against the inherently dark and noisy background of rusted pipes under low-light conditions. This results in weak spatial gradient signals. During the deep convolutional downsampling processes, these fragile, fine-grained boundary features are easily attenuated or entirely lost. This theoretical insight not only explains the current model’s failure modes but also underscores the necessity for integrating enhanced contrast-aware mechanisms and high-resolution feature preservation strategies in future pipeline inspection models.

Table 5 presents the experimental results from optimizing the YOLO-LSS model on the validation dataset. The findings indicate that the penetration category exhibits the best performance, achieving a high precision of 92.2%, a recall rate of 92.9%, and an excellent F1-score of 92.3%. Additionally, it demonstrates commendable results in both mAP50 and mAP50:95 metrics, highlighting its outstanding detection efficacy. In contrast, the detection performance is slightly inferior to that of the penetration category for the corrosion, deposition, and oxide shedding categories. This discrepancy may be attributed to the complexity of defects or insufficient samples for these types of defects, leading to inadequate feature extraction. Nevertheless, it is noteworthy that significant improvements are observed in the detection of defects with the improved model in all four categories.

**Figure 10:**
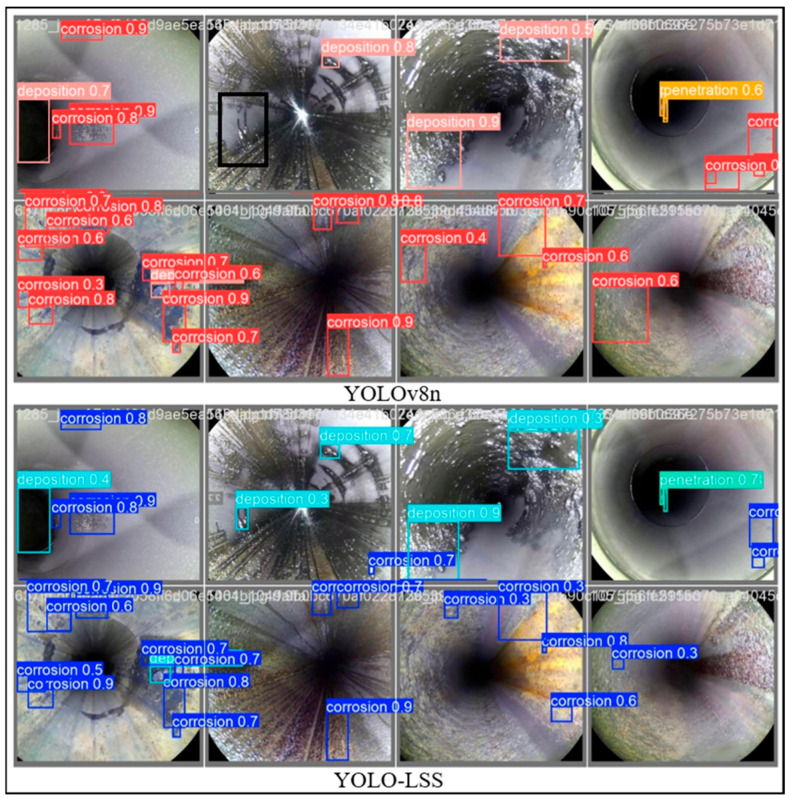
Comparison of detection between the original YOLOv8n and the YOLO-LSS model.

Figure 10 indicates that the original YOLOv8n model may miss defects, as illustrated in the black box regions (This part was not detected in the YOLOv8n model, so it was marked with a black box.) during the detection process. In contrast, the YOLO-LSS model successfully identifies these defects. Furthermore, the optimized model shows a significant improvement in confidence levels in the detection of defects compared to the original model. This improvement is attributed to the incorporation of multiple modules in this experiment, which improves the model’s ability to recognize features associated with different defects. Although the YOLO-LSS model, empowered by the SCSA and LskBlock modules, significantly enhances the detection accuracy for small-sized targets compared to the original YOLOv8n, extreme micro-defects still pose one of the most significant challenges among all defect categories due to severe environmental noise. Therefore, further exploring techniques such as advanced multi-scale feature fusion or super-resolution specifically for these extreme edge cases remains a valuable direction for future studies.

**Figure 11:**
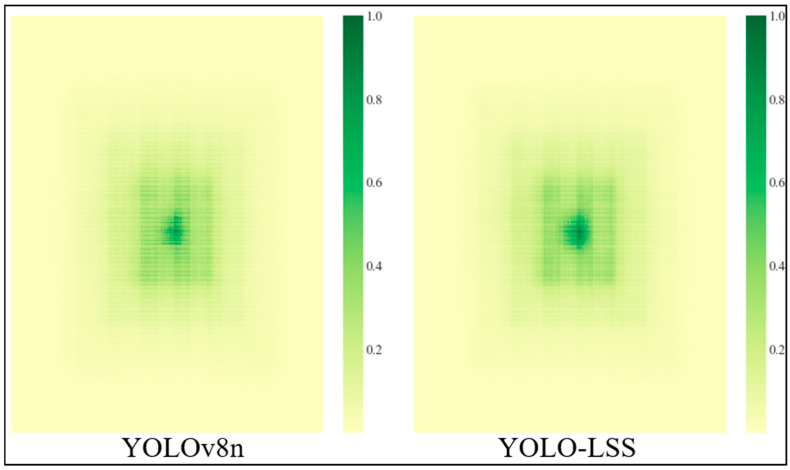
Effective receptive fields of different models.

Figure 11 presents a comparative analysis between YOLOv8n and the improved model regarding the effective receptive fields (ERFs). A random selection of 500 training images is tested. A larger ERF may better capture the global information of the images. As depicted in Figure 10, the improved model possesses a broader perceptual range than its predecessor, indicating that the YOLO-LSS framework extracts more comprehensive and accurate information than the original YOLOv8n model.

**Figure 12:**
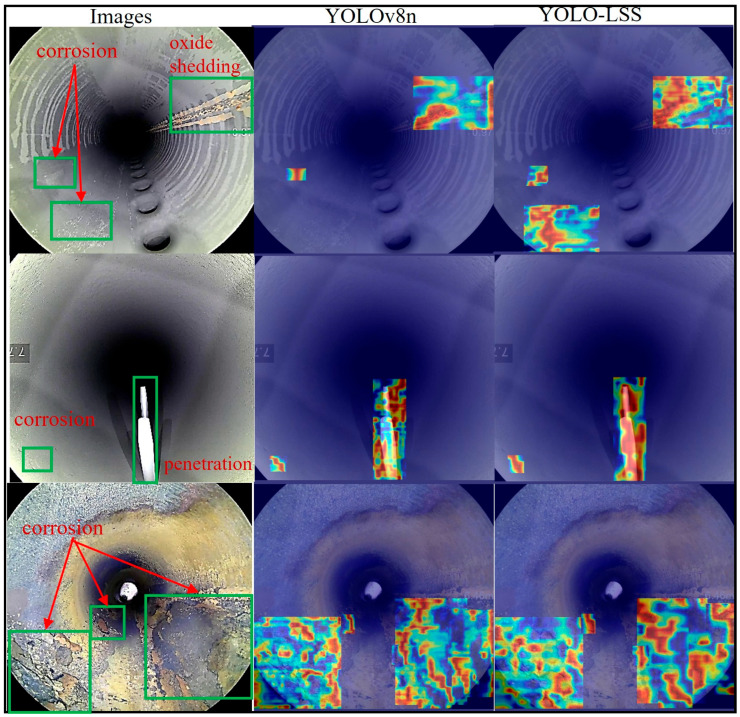
Heatmaps of different models.

This study employs heatmap visualization to analyze and compare decision-making mechanisms between the modified YOLO-LSS and original YOLOv8n models (see Figure 12). Spatial focusing strategies are used to eliminate background noise interference in the experiment. The heatmap analysis is limited to bounding box regions, which may precisely interpret the effectiveness of the model to capture the features of the defects. Based on the GradCAMPlusPlus framework, both models generate saliency maps capable of capturing core defect areas (highlighted in red). Furthermore, activation distributions are more compact for the improved model within bounding boxes, suggesting that YOLO-LSS model has better accuracy than the original YOLOv8n model.

### 4.3. Statistical Analysis

Table 6 presents the mAP50 results from three independent experimental runs (Run 1 to Run 3) for each model, along with their mean and Standard Deviation (SD). The data demonstrate that the proposed YOLO-LSS model achieves the highest mAP50 (79.05%) and the lowest variance (SD = 0.13), indicating its superior detection stability and statistical reliability compared to other models.

### 4.4. Comparative Experiment

To further evaluate the detection advantages of the YOLOv8n-LskBlock-SlideLoss-SCSA (YOLO-LSS) model for pipeline defects, this study conducted comparative experiments with other mainstream object detection algorithms on the experimental dataset. The primary baseline models include Faster-RCNN, YOLOv3, YOLOv5s, YOLOv8, YOLOv9, YOLOv10, SSD, and RT-DETR. To ensure the accuracy and objectivity of the experimental evaluation, the training configurations for all comparative architectures must be explicitly defined. This study evaluated the models under a strictly controlled software and hardware environment. All models were trained using the exact same dataset splits and the identical data augmentation pipeline as the proposed model. Given the distinct optimization characteristics of different network topologies, we did not enforce uniform hyperparameters. Instead, architecture-specific hyperparameters—such as the optimizer (e.g., Adam, SGD, AdamW), initial learning rate, weight decay, optimal input resolutions, and training epochs—were set to the optimal default values recommended by their respective foundational implementations. Furthermore, for traditional models like Faster-RCNN and SSD, considering their specific convergence characteristics, we followed standard training protocols and set the training duration to 100 epochs, which is experimentally sufficient to ensure full convergence for these architectures. This strategy ensures that each comparative model is evaluated at its maximum potential, thereby providing a highly rigorous comparative baseline. The detailed training hyperparameters for each evaluated model are summarized in Table 7.

Table 8 summarizes the performance of some network models in this comparative experiment, with a specific focus on analyzing the trade-off between detection accuracy and computational efficiency—a critical factor determining the feasibility of industrial edge deployment. In terms of detection accuracy, the YOLO-LSS model achieved the highest scores across most evaluation metrics. We objectively acknowledge that compared to the recent RT-DETR model, the performance improvement of our model is relatively marginal (e.g., the mAP50:95 of YOLO-LSS is 53.7%, whereas RT-DETR achieves 51.8%). However, this competitive detection performance must be evaluated in conjunction with model complexity. As shown in Table 8, RT-DETR, a heavy Transformer-based architecture, requires 124 MB of storage, while the proposed YOLO-LSS model requires only 7.05 MB. Achieving a slightly higher detection accuracy while maintaining an extremely lightweight computational footprint (nearly 18 times smaller than RT-DETR) robustly validates the efficiency of the proposed architectural improvements. Regarding real-time performance and latency, the YOLO-LSS model achieves an inference speed of up to 188 Frames Per Second (FPS), which translates to a low inference latency of approximately 5.32 milliseconds per image. This speed exceeds the standard video stream processing requirement of 30 FPS, effectively satisfying the real-time demands of practical industrial operations.

Finally, regarding the internal complexity cost analysis, the integration of the SCSA mechanism, LskBlock, and SlideLoss function inevitably increased the model’s complexity slightly compared to the original YOLOv8n baseline. Specifically, the model size marginally increased from 6.09 MB to 7.05 MB, and the inference speed experienced a minor drop from 202 FPS to 188 FPS. Nevertheless, this negligible increase in computational cost is a highly worthwhile trade-off for the notable enhancement in detection accuracy (with mAP50:95 increasing from 51.4% to 53.7%). Such a lightweight profile guarantees an extremely low hardware threshold, making the YOLO-LSS model highly suitable for physical deployment on resource-constrained industrial edge devices, such as mobile pipeline inspection robots, thereby practically fulfilling the needs of industrial production.

### 4.5. The Superiority of the Developed Model

The proposed AM is compared with others, including the MSAM (Multi-Scale Attention Module [43]), CPCA (Channel Prior Convolutional Attention [44]), and GAM (Global Attention Mechanism [45]). The comparison is presented in Table 9. The SCSA exhibits better integration with the enhanced model, and generates higher metrics (precision (P), recall (R), F1-score, and mAP50-95) than the other AMs. Specifically, the YOLO-LSS model improves by 6.81% in accuracy, 7.09% in recall rate, 6.14% in F1-score, and 9.82% in mAP50-95 compared to the YOLOv8n-LskBlock-SlideLoss-MSAM model. Similarly, the corresponding improvements are 1.24%, 8.17%, 4.01%, and 4.68% when the YOLO-LSS model is compared with the YOLOv8n-LskBlock-SlideLoss-CPCA model. Furthermore, the YOLO-LSS model has a higher accuracy of 4.48%, a higher recall rate of 4.28%, a higher F1-score of 3.6%, and a higher mAP50-95 of 3.07% than the YOLOv8n-LskBlock-SlideLoss-GAM model. Thus, the SCSA is highly effective for detecting the defects of pipelines in low-light or dimly lit scenarios.

### 4.6. Ablation Experiment

The ablation experiments are conducted to validate the effectiveness of the improved model. Eight experiments are taken and the results are summarized in Table 10, where S denotes the SCSA module, SL represents the improved loss function, and LS refers to the LskBlock module. One may see that the improved model demonstrates the best performance in terms of accuracy, recall, mAP50-95, and F1-score in the eight experiments. Each partial model is crucial to the improved model. The absence of the partial models leads to a decline in the overall performance of the improved model. The accuracy increases by 4.39% when SCSA is added to the original YOLOv8n model. In addition, the introduction of the LskBlock module increases the complexity of the model. This is because LSKNet is a large selective kernel network. However, improvements are also noted in various metrics, such as precision and recall.

### 4.7. Comprehensive Comparison with Mainstream and Transformer-Based Methods

To further evaluate the engineering application potential of the proposed model, Table 11 presents a comprehensive comparison between YOLO-LSS, mainstream lightweight YOLO variants, and recent Transformer/DETR architectures in terms of computational overhead, detection accuracy, and application limitations.

As shown in Table 11, existing lightweight networks (e.g., Z. Li et al.) typically focus on a single defect category, exhibiting relatively limited generalization capabilities in complex environments. Conversely, while recent industrial-grade models (e.g., AM-DETR by Z. Lv et al.) achieve high detection accuracy, their relatively large parameter scale (22.34 M) and sensitivity to extreme illumination conditions increase the difficulty of deployment on edge devices such as those in underground pipelines. In contrast, the proposed YOLO-LSS model achieves real-time inference at 188 FPS with a compact model size of 7.05 MB, effectively mitigating computational bottlenecks. These results demonstrate that the proposed model strikes a favorable balance among detection accuracy, inference speed, and hardware resource consumption, effectively accommodating the real-time physical deployment requirements of pipeline robots.

## 5. Conclusions

The ability to identify multiple defects in complex environments is crucial for the sustainable development of the metal pipeline industry. This study proposes an improved model based on the original YOLOv8n architecture to detect defects in buried pipelines. Based on the extensive investigations, the main conclusions are drawn below:

(1) The SCSA module is incorporated to identify defects in complex environments.
(2) The neck structure, LskBlock, improves the detection accuracy by dynamically adjusting the receptive field for each target.
(3) Experimental results indicate that the proposed model achieves an mAP50:95 of 53.7%, representing an improvement of 7.19% over the original model.
(4) This study provides efficient new algorithms for detecting internal defects of pipelines in complex and low-light environments.

Furthermore, to address the model’s current weakness in detecting small-sized targets, future studies will explore the integration of advanced techniques such as super-resolution, feature pyramid enhancement, or segmentation-assisted detection approaches to further improve the detection accuracy for minor defects. Additionally, there are some limitations in the current evaluation regarding dataset diversity and independent robustness testing. Although the images were collected directly from actual pipeline engineering environments and already contain common inspection conditions, the model was primarily validated on a single dataset. Therefore, conclusions regarding its broad generalization capability and robustness in entirely unseen or highly distinct pipeline environments remain preliminary. Due to time constraints in the current research phase, further dataset expansion and testing across varying, incremental noise levels were not performed at this stage. Future work will focus on collecting a more comprehensive dataset under extreme conditions and designing a systematic robust evaluation framework to further optimize the model’s practical performance.

## Figures and Tables

**Table 1:** Dataset segmentation.

Type of Dataset	Quantities	Proportions
training set	7752	93.7%
validation set	259	3.15%
Test set	260	3.15%

**Table 2:** Software and hardware parameters.

Hardware	Description	Software	Description
GPU	GeForce RTX 3070Ti	Operating system	Windows 10
		Deep Learning Framework	PyTorch
CPU	13th Gen Intel(R) Core (TM) i5- 13490F @2.50 GHz	CUDA	12.1
		Python	3.8

**Table 3:** Training parameters.

Parameter Name	Parameter Settings
Momentum	0.937
Input Image Size	640 × 640
Number of Training Epochs	300
Batch Size	16
Optimizer	Adam
Learning Rate	0.01

**Table 4:** Refinement of benchmark algorithms before experiments.

Model Name	P/%	R/%	mAP50-95/%	GFLOPS	Size (MB)
YOLOv8n	77.4	72.4	50.1	8.2	6.3
YOLOv8s	83.8	78.5	55.8	28.7	22.6
YOLOv8m	86.4	79.8	63.4	79.0	50.3
YOLOv8l	86.7	79.6	63.3	162.5	86.5
YOLOv8x	88.1	80.3	64.7	251.2	128

**Table 5:** Experimental results of the YOLO-LSS model based on the valid dataset.

Class	P/%	R/%	F1-Score	mAP50/%	mAP50:95/%
All	81.5	74.5	77.8	79	53.7
Corrosion	83.7	57	67.8	68.5	45.5
Deposition	70.9	74.5	72.7	73.9	55.9
Oxide shedding	79.4	73.5	76.3	79.3	50.6
Penetration	92.2	92.9	92.3	94.5	62.6

**Table 6:** Statistical results of mAP50 across three independent runs.

Model	mAP50/%				
	Run 1	Run 2	Run 3	Mean	SD
Ours	79	79.2	78.95	79.05	0.13
YOLOv3	77.6	77	77.28	77.29	0.3
YOLOv5s	77.9	78.32	78.13	78.12	0.21
YOLOv8	78	77.56	78	77.85	0.25
YOLOv9	78.2	78.55	78.31	78.35	0.18
YOLOv10	77.5	77.12	77.34	77.32	0.19
SSD	76.3	77.1	76.74	76.71	0.4
Faster-RCNN	75.8	74.84	75.36	75.33	0.48
RT-DETR	78.8	79.1	78.93	78.94	0.15

**Table 7:** Training parameters for the comparative models.

Model	Optimizer	Learning Rate	Momentum	Input Image Size	Epochs	Batch Size	Weight Decay
YOLOv3	Adam	0.01	0.937	640 × 640	300	16	0.0005
YOLOv5s	Adam	0.01	0.937	640 × 640	300	16	0.0005
YOLOv8	Adam	0.01	0.937	640 × 640	300	16	0.0005
YOLOv9	Adam	0.01	0.937	640 × 640	300	16	0.0005
YOLOv10	Adam	0.01	0.937	640 × 640	300	16	0.0005
SSD	SGD	0.001	0.900	300 × 300	100	8	0.0005
Faster-RCNN	SGD	0.005	0.900	800 × 800	100	4	0.0001
RT-DETR	AdamW	0.0001	0.900 (β1)	640 × 640	300	16	0.0001

**Table 8:** Comparison of different models on the test set.

Model	Precision/%	Recall/%	F1-Scores	mAP50/%	mAP50/% ± SD	mAP50:95/%	FPS	Size
Ours	81.6	75.5	78.4	79.05	79.05 ± 0.13	53.7	188	7.05 MB
YOLOv3	78.5	73.7	76.0	77.29	77.29 ± 0.30	48.1	146	127 MB
YOLOv5s	80.2	74.0	77.0	78.12	78.12 ± 0.21	49.2	183	14 MB
YOLOv8	80.5	74.3	77.3	77.85	77.85 ± 0.25	51.4	202	6.09 MB
YOLOv9	80.9	74.8	77.7	78.35	78.35 ± 0.18	52.1	210	5.74 MB
YOLOv10	81.4	73.2	77.1	77.32	77.32 ± 0.19	49.3	235	5.80 MB
SSD	63.7	61.6	62.6	76.71	76.71 ± 0.40	45.8	47	95 MB
Faster-RCNN	52.4	58.7	55.4	75.33	75.33 ± 0.48	44.1	25	162 MB
RT-DETR	81.1	75.1	78.0	78.94	78.94 ± 0.15	51.8	164	124 MB

**Table 9:** Comparative analysis of attention mechanism performance (test dataset).

Model	P/%	R/%	mAP50-95/%	F1-Score
YOLOv8n-LskBlock-SlideLoss-SCSA	81.6	75.5	53.7	78.4
YOLOv8n-LskBlock-SlideLoss-MSAM	76.4	70.5	48.9	73.3
YOLOv8n-LskBlock-SlideLoss-CPCA	80.6	69.8	51.3	74.8
YOLOv8n-LskBlock-SlideLoss-GAM	78.1	72.4	52.1	75.1

**Table 10:** Ablation results of the proposed model.

S	LS	SL	Parameters	GFLOPS	P/%	R/%	mAP50-95/%	F1-Score
×	×	×	3,011,628	8.2	77.4	72.4	50.1	74.8
√	×	×	3,212,076	8.4	80.8	72.9	53.3	76.6
×	√	×	3,349,118	8.5	79.3	73.6	52.5	76.3
×	×	√	3,011,628	8.2	79.2	72.8	51.0	75.9
√	√	×	3,349,566	8.5	81.3	73.1	52.9	77.0
√	×	√	3,212,076	8.4	81.1	72.4	51.7	76.5
×	√	√	3,349,118	8.5	80.6	72.3	52.5	76.2
√	√	√	3,349,566	8.5	81.5	74.5	53.7	77.8

**Table 11:** Comprehensive performance comparison of the proposed YOLO-LSS models with existing methods.

Study	Method	Application Scenario	Real-Time Capability/Cost	Accuracy	Limitation
Yin et al.	SPVA System (YOLOv3)	Sewer pipe CCTV videos	~33 FPS/Offline process	mAP50: 85.37%	- Designed primarily for off-site video assessment. - Prone to noise and false alarms requiring extra filtering.
Zhao et al.	Optimized YOLOv8	Pipeline inner wall (corrosion)	Yes (10.0 GFLOPS, 5.81 MB)	mAP50: 95.0%	- Weak in detecting small-sized defect targets. - Focused exclusively on a single corrosion category.
Z. Li et al. [46]	YOLOv8-GhostConv-SEV2	Buried pipeline cracks (low-light)	Yes (5.67 MB)	mAP50: 99.2%	- Focuses exclusively on single-class crack detection. - Needs future extension to handle multi-class interference like paint peeling or rust spots.
Chen et al.	EFS-YOLO	Steel strip surface	Yes (>100 FPS estimated)	increased by 2.4%	- Designed for 2D flat surfaces. - Less adaptable to cylindrical depths and water reflections.
P. Chen et al. [47]	Cascaded YOLOv5 + ViT	Pipeline defects (MFL signals)	No (<1 FPS, takes “seconds”)/~88 M Params	Precision: 96.75%	- Heavy Transformer architecture leads to severe latency. - Relies on specific magnetic sensors (not standard CCTV).
Z. Lv et al. [48]	AM-DETR	Photovoltaic cell surface defects	Yes (156.3 FPS)/22.34 M Params, 64.3 GFLOPS	mAP50: 92.2%	- Parameter scale and GFLOPS are still relatively heavy for extreme nano-edge devices. - Vulnerable to extreme illumination conditions (unsuitable for dark underground pipes).
This Work	YOLO-LSS	Underground metal pipelines	Yes (188 FPS, 7.05 MB)	mAP50: 79.05%	- Relatively lower recall for oxide shedding class. - Relies on supervised learning with annotated data.

## Data Availability

All data, models, and code generated or used during the study appear in the submitted article.
